# MFDA: Multiview fusion based on dual-level attention for drug interaction prediction

**DOI:** 10.3389/fphar.2022.1021329

**Published:** 2022-10-06

**Authors:** Kaibiao Lin, Liping Kang, Fan Yang, Ping Lu, Jiangtao Lu

**Affiliations:** ^1^ School of Computer and Information Engineering, Xiamen University of Technology, Xiamen, China; ^2^ Shenzhen Research Institute of Xiamen University, Shenzhen, China; ^3^ Department of Automation, Xiamen University, Xiamen, China; ^4^ School of Economics and Management, Xiamen University of Technology, Xiamen, China

**Keywords:** multiview, dual-level attention, cross-fusion strategy, graph attention network, drug-drug interactions

## Abstract

Drug-drug interaction prediction plays an important role in pharmacology and clinical applications. Most traditional methods predict drug interactions based on drug attributes or network structure. They usually have three limitations: 1) failing to integrate drug features and network structures well, resulting in less informative drug embeddings; 2) being restricted to a single view of drug interaction relationships; 3) ignoring the importance of different neighbors. To tackle these challenges, this paper proposed a multiview fusion based on dual-level attention to predict drug interactions (called MFDA). The MFDA first constructed multiple views for the drug interaction relationship, and then adopted a cross-fusion strategy to deeply fuse drug features with the drug interaction network under each view. To distinguish the importance of different neighbors and views, MFDA adopted a dual-level attention mechanism (node level and view level) to obtain the unified drug embedding for drug interaction prediction. Extensive experiments were conducted on real datasets, and the MFDA demonstrated superior performance compared to state-of-the-art baselines. In the multitask analysis of new drug reactions, MFDA obtained higher scores on multiple metrics. In addition, its prediction results corresponded to specific drug reaction events, which achieved more accurate predictions.

## Introduction

Drug combination therapy is widely used in clinical practice and has been recognized as a safe and effective treatment for serious diseases (Sun et al., 2016). However, multiple medications can also lead to harmful drug-drug interactions (DDIs), which can be life-threatening in severe cases and lead to the withdrawal of drugs from the market. The methods currently available to detect DDIs are primarily through long-term *in vivo* and *in vitro* clinical trials, which are costly and tedious. Therefore, it is critical to propose an efficient method for DDI identification.

**FIGURE 1:**
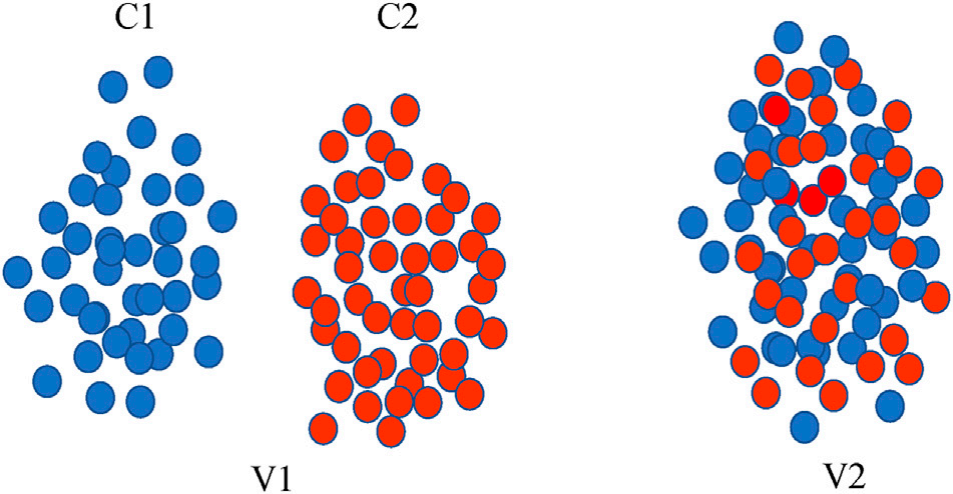
Two different views. Cl and C2 denote two categories in two different colors. V1 and V2 represent two views, in view V1, the two categories can be easily distinguished by the classifier, while in view V2, the data of these two categories are mixed and difficult to separate.

Nowadays, AI-based computational methods have been widely used in biomedical fields (Dong et al., 2022; Meng et al., 2022), such as drug repositioning (Pan et al., 2022), protein interactions (Song et al., 2022), and DDI predictions (Deng et al., 2020). For DDI prediction, feature or topological similarity-based approaches are the most common. Many methods have been developed based on these methods in different ways, including aggregating multiple types of drug features (Gottlieb et al., 2012), predicting multi-class drug reaction events (Ryu et al., 2018), and integrating drug attribute features with drug interaction relationship (Kang et al., 2022). These optimized methods are effective and make the DDI predictions more complete. However, most of those methods ignore the importance of multiview learning (Blum and Mitchell, 1998) and do not take it into consideration. In recent years, multiview learning has been successfully applied to many tasks. As illustrated in Figure 1 for the classification task, integrating multiple views can capture more discriminative features and thus enhance difference. It means that integrating multiple views facilitates to capture of more comprehensive relationships. The situation also applies to drug interaction networks, as the reactions between drugs are complex and diverse. The adjacency view (graph), which is often used in experiments, reflects only one aspect of direct similarity between interacting drugs. Indeed, similarities between nodes that do not directly interact have proven to be very useful in biological networks (Huang et al., 2020), including genetic interactions and protein-protein interaction networks (Costanzo et al., 2016; Kovács et al., 2018). Therefore, we propose a novel multiview fusion approach (MFDA) to predict DDI in this paper. In addition to the adjacency view that provides local topological relations, we add a diffusion view providing beyond first-order topological relationships and the nearest neighbor view constructed from feature similarities to capture a more comprehensive and accurate DDI pattern. Moreover, for the integration of drug features and network structures under each view, we employed cross-fusion to flexibly exchange and fuse the two sources of information. Our contributions are summarized as follows:

1) This study presents a novel MFDA method to predict drug interactions. It constructs multiple relational views of drug interactions and employs a cross-fusion strategy to handle the fusion of drug features and topological information under each view. The experiment results prove the effectiveness of the proposed method.
2) MFDA proposes a dual-level attention mechanism to distinguish the importance of drug nodes and different views for effective fusion. Furthermore, the attention score can also provide interpretable predictions for drug reactions.
3) Most traditional methods usually focus on binary prediction results, and MFDA can predict multiple drug reaction events, which is more useful for investigating the mechanism hidden behind the drug reactions.

### Related work

Many computation-based methods have been proposed for DDI prediction, and they can be mainly divided into the following four groups.

Feature similarity-based methods assume that drugs with similar features share similar reaction patterns. In early DDI studies, researchers focused on the calculation of drug feature similarities for DDI prediction, such as side effects (Tatonetti et al., 2011), chemical structure (Vilar et al., 2012), and phenotype (Peng et al., 2015). Later, multiple features were combined to improve the model performance (Gottlieb et al., 2012; Cheng and Zhao, 2014; Yan et al., 2020). For example, Kastrin et al. (2018) considered semantic and topological similarities and adopted five classifiers to build a prediction model. Yan et al. (2019) proposed IDNDDI, which used cosine similarity to calculate information about chemical structure, biological and phenotypic features of drugs to infer drug reactions. Rohani and Eslahchi. (2019) calculated multiple drug similarities and used a heuristic similarity selection method to select features that are informative and less redundant for combination. These methods are natural and straightforward, but they ignore structural information. In addition, the selection of features relies on expert experience.

Graph embedding-based methods automatically learn low-dimensional node embeddings from relational networks and use them as node features. For example, Yue et al. (2020) proposed a common graph representation learning framework for predicting drug reactions. It utilized three common types of graph embedding methods (factorization, random walk, and neural networks) to obtain drug embeddings that were used to infer potential drug reactions. The above approaches follow an unsupervised general framework that leads to suboptimal model performance. Subsequent efforts were made to improve it for specific DDI tasks. For example, to obtain high-order drug topological relations, SkipGNN (Huang et al., 2020) aggregated neighboring topological information within two hops for DDI prediction. To introduce multiple drug-related entities, Yan et al. (2021) constructed five drug-related heterogeneous networks and implemented random wander with restart algorithms and positive point mutual information to obtain unified topological embeddings for prediction. The above optimized methods are promising, but they fail to take feature information into consideration, which can also provide discriminative information for prediction tasks.

Attribute-based network methods combine both network topology and attribute information to obtain more comprehensive information. They are usually optimized by incorporating drug feature information into previous graph embedding methods. For example, among the factorization-based approaches (Xiao et al., 2017; Yu et al., 2018; Zhu et al., 2020), Zhu et al. (2020) proposed an attribute supervised learning model that incorporated two drug attributes, molecular structure and side effects, and their correlation to infer adverse interactions among drugs. In graph neural network approaches (Zitnik et al., 2018; Kip F and Welling, 2017), where propagation-based methods usually incorporate the feature information into the relational network. For instance, Zitnik et al. (2018) proposed Decagon, which constructed a multimodal network and adopted convolutional operators to propagate and transform feature vectors across the multirelational network. As a result, the final obtained embedding vector learned both the drug attribute information and the structure information. Decagon has achieved favorable results by encoding feature information of multiple entities. To easily integrate multiple entities and handle multiple relationships flexibly, knowledge graph (KG)-based methods have received more attention (Zeng et al., 2022). Many excellent models have been proposed, such as the relation-aware network embedding model for DDI prediction (RANEDDI) (Hui et al., 2021), multimodal deep neural network (MDNN) (Lyu et al., 2021), and knowledge summarization GNN (Sum-GNN) (Yu et al., 2021), which can better capture the structure and semantic information of the network.

The above attribute-based network methods have made considerable improvements over pure structure-based methods, but they do not integrate the information about attributes and structures well. For example, factorization-based methods are shallow models that cannot capture the nonlinear relationship between the two information sources. Propagation-based methods prefer network structures to drug attributes and may result in oversmoothing problems. More importantly, these methods mainly focus on a single view of drug reaction relationships and ignore the importance of multiple views. As a result, the model’s results are highly dependent on the quality of the selected view, resulting in poor robustness.

Multiview-based methods construct multiple views of the same target and learn by exploiting the complementarity complementary between views. Many multiview learning methods have been proposed. For example, Wang et al. (2020) proposed AM-GCN, which constructed two views from network structure and node features, respectively, and then used an attention mechanism to automatically learn the weights of the views. Yuan et al. (2021) designed three complementary views and adopted convolutional operations to learn view embeddings. Finally, an attention mechanism was also used to fuse node representations for the classification task. Wang et al. (2021) proposed to capture inter-view molecule structure and intra-view interactions between drugs, and then used an unsupervised contrast learning component to balance and integrate multiview information. Although the above methods are effective, there is room for improvement. For instance, AM-GCN only constructed two views, and Yuan et al. (2021) used convolution operations to simply aggregate node features and topological information, which limits the model to capture important information. Therefore, we constructed three views to learn the comprehensive drug interaction relationships. Unlike before, we propose a cross-fusion strategy to fuse drug features and topologies under each view. Specifically, it utilizes dual channels to encode drug features and network structures separately and then exchanges information flexibly with convey operations. Through extensive experiments, the experimental results show that our model outperforms other optimal methods.

## Methodology

### Overview

**FIGURE 2:**
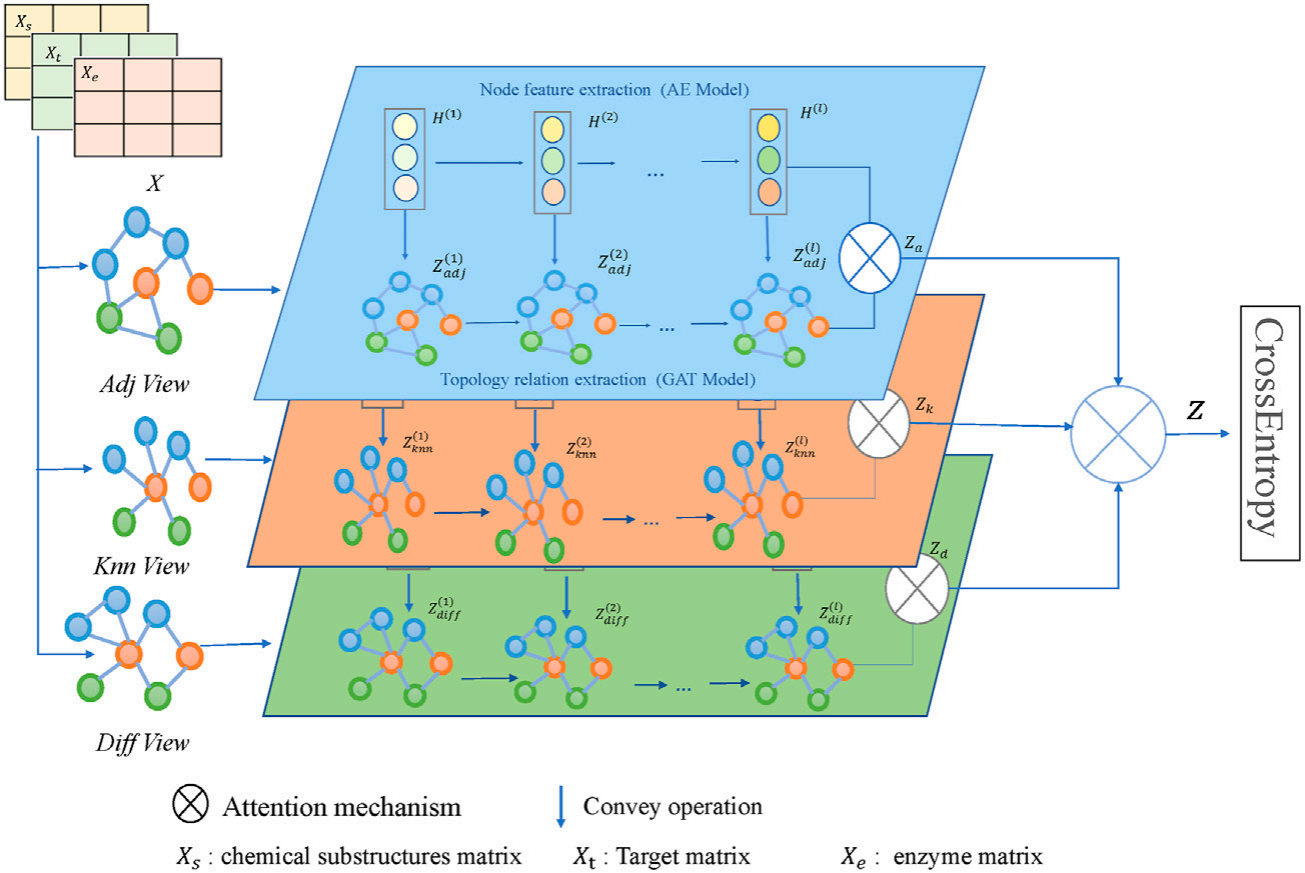
Illustration of the MFDA framework.

The framework of the model MFDA is shown in Figure 2. It consists of four main parts: multiple-view construction, dual-level attention mechanism, cross-fusion strategy, and model optimization. The model first constructed three graphs $A_{adj}$ , $A_{diff}$ , and $A_{knn}$ from different views. Under each view, the type-specific graph $A_{y}$ and feature matrix X were input into corresponding networks. To effectively combine the two sources of information, a cross-fusion strategy was employed in this paper. Specifically, the autoencoder (AE) was used on the drug feature matrix to extract feature information, while the graph attention network (GAT) was applied to capture structural information from the adjacency matrix. Then, we adopted convey operations to fuse the two types of information between each layer. After multiple iterations, three graph representations $Z_{adj}$ , $Z_{diff}$ , and $Z_{knn}$ are available. Then they are fused using the attention mechanism module to obtain a unified drug representation Z . Finally, multiple drug pair vectors were constructed and fed into the loss function for training and optimization.

### Problem definition

In this section, we use the relevant notation in Table 1 to state the problem definition. Given a multirelational DDI network G={V,E,X} , where the vertices V denote the drug nodes, the edges E denote the types of drug reactions. We also have feature matrix X = ${\left[X_{1},X_{2}\ldots X_{i}\right]}^{T}\in R^{N}$ , where N denotes the dimensions of the feature vector, and each row in the matrix corresponds to a drug feature vector. The main task of the MFDA model is to predict the specific type of reaction between drugs, which is essentially defined as a multiclassification problem. The prediction results represent the probability distribution over multiple DDI types, and the highest score is used as the final prediction type.

**TABLE 1:** The main notation and definition.

Notation	Definition and description	Notation	Definition and description
G	The input network	Z	The unified embedding
V	The node set	$Z_{y}$	The graph embedding learned in view y
E	The edge set	$Z_{y}^{\left(l\right)}$	The embedding learned by GAT in view y at layer l
X	The final attributes feature of all nodes	$H^{\left(l\right)}$	The embedding learned by AE at layer l
$X_{i}$	The final attribute feature of drug i	$\varepsilon_{y}$	The attention coefficients in view y
$\alpha_{ij}$	The weight coefficient of neighbor j on i	$A_{y}$	The adjacency matrix constructed in y view
$X_{x}$	The similarity feature matrix on feature x	$s_{ij}$	The feature similarity of drug i,j vectors

### Multiple view construction

Given a graph G , we constructed three widely-studied graphs from different views, which represent different structures extracted from the drug relationship network.

1) Adjacency graph (denoted $A_{adj}$ ), which reflects the local structure. If two drugs interact, the corresponding position in the $A_{adj}$ matrix is replaced with a “1”; otherwise, it is replaced with a “0”.
2) Diffusion graph (denoted $A_{diff}$ ), which provides a global view of the drug interaction relationship. It can capture information beyond first-order neighbors to predict DDI. We use the Personalized PageRank (PPR) by Eq. 1 to obtain the diffusion graph.

$$A_{diff}=\alpha {\left(I-\left(1-\alpha \right)D^{-\frac{1}{2}}AD^{-\frac{1}{2}}\right)}^{-1}$$ (1)

where α∈ (0, one) denotes the transition probability in a random walk, I is an identity matrix, and D is the degree matrix of A .

3) K-nearest neighbors graph (denoted $A_{knn}$ ), reflects the similarity of the feature space. First, for each drug, we select its top-K similar neighbors by computing the cosine similarity using Eq. 2, and here set the neighbor sampling size K = 2.

$$s_{ij}=\frac{X_{di}∙X_{dj}}{∥X_{di}∥∥X_{dj}∥}$$ (2)

where $X_{di}$ and $X_{dj}$ denote the feature vectors of drug i and j . The symbol ∥ indicates the Euclidean norm operation. $s_{ij}$ indicates the similarity between two vectors i and j . After that, we connect an edge between nodes and their first ten neighbors, so the KNN graph is constructed.

### Cross fusion strategy

To effectively fuse the network structure and node attribute information of each view, this study adopts a cross-fusion strategy.

First, we need to process the three raw features given in this study (substructure, target, enzyme). Since each feature corresponds to a set of feature descriptors, and each drug can be represented by a binary vector, we calculate the pairwise similarity by performing the Jaccard similarity using Eq. 3.

$$J\left(di,dj\right)=\frac{\left|di\cap dj\right|}{\left|di\cup dj\right|}=\frac{\left|di\cap dj\right|}{\left|di\right|+\left|dj\right|-\left|di\cap dj\right|}$$ (3)

where di and dj represent bit vectors drugs i and j , respectively; di∩dj is the intersection and di∪dj is the union. After the Jaccard calculation, we can obtain the similarity matrix of substructure, target, and enzyme features, which are represented as $X_{s},X_{t},X_{e}$ respectively. Then, they are concatenated as the unified feature matrix X , where the feature vector of drug i is defined as Eq. 4.

$$X_{i}=X_{s}^{i}⊕X_{t}^{i}⊕X_{e}^{i}$$ (4)

We use dual channels to encode drug features and network structures separately. For clarity, we take drug feature X and the adjacency graph $A_{adj}$ as an example.

For drug features, we adopt the widely used AE to learn feature embeddings. $H^{\left(l\right)}$ denotes the feature embedding learned at the $l_{th}$ layer, as defined in Eq. 5.

$$H^{\left(l\;\right)}=\emptyset \left(W_{e}^{\left(l\right)}H^{\left(\;l-1\right)}+b_{e}^{\left(l\right)}\right)$$ (5)

where ∅ is the ReLU activation function, and W and b are the weight matrix and bias matrix at the $l_{th}$ layer, respectively. $H^{\left(0\right)}$ denotes the feature matrix X .

For the network structures, different neighbors play various roles for the central node. Therefore, we use a node-level attention mechanism to assign associated weights to neighbors, which is essentially implemented through GAT. The topological embedding learned at each layer is denoted $Z_{adj}^{\left(1\right)}$ , $Z_{adj}^{\left(2\right)}$ , … , and $Z_{adj}^{\left(l\right)}$ . We set up convey operations to allow the two channels to exchange and fuse information flexibly. That is, the feature embedding captured by AE is conveyed to the GAT with a certain ratio ε . The formal formulation is shown in Eq. 6.

$${\tilde{Z}}_{adj}^{\left(l-1\right)}=\left(1-\varepsilon \right)Z_{adj}^{\left(l-1\right)}+\varepsilon H^{\left(l-1\right)}$$ (6)

ε is the fusion coefficient, which balances the importance of two learned vectors from the GAT and the AE. Then, ${\tilde{Z}}_{adj}^{\left(l-1\right)}$ is fed into the next GAT layer to obtain the fused representation, as shown in Eq. 7.

$$Z_{adj}^{\left(l\right)}=\emptyset \left({\tilde{D}}^{-\frac{1}{2}}\tilde{A}{\tilde{D}}^{-\frac{1}{2}}{\tilde{Z}}_{adj}^{\left(l-1\right)}W^{\left(l-1\right)}\right)$$ (7)

where ∅ represents the ReLU activation function and $Z_{adj}^{\left(l\right)}$ is the fused embedding of the GAT channel at the $l_{th}$ layer. To maximize the use of information from both channels, the two learned embeddings are finally fused *via* an attention mechanism module, which is similar to the view-level attention mechanism (refer to *View-level attention mechanism*). As a result, three view-level embedding vectors $Z_{adj}$ , $Z_{diff}$ , and $Z_{diff}$ can be obtained. The three embeddings are informative as they accommodate information about drug features and topological relationships of the first l layers. The high-level embedding representation lays a strong foundation for downstream tasks.

### Dual-level attention mechanism

The influence of different neighbors on the central node is distinct, as are the different views. As an analogy, it seems that when evaluating a person, his or her parents, friends, classmates, and teachers (regarded as the node level) do not have the same opinions about him. Notably, parents and friends have better familiarity, and their judgment should be more comprehensive. Considering other aspects, such as learning and sociability (regarded as the view level), the impact of their evaluation should also be distinguished. This phenomenon may also apply to the prediction of drug reactions. Therefore, we use a dual-level attention mechanism to assign learnable weights to different nodes and views.

#### Node-level attention mechanism

Considering that the importance of different neighbor nodes is varied, we adopt a self-attention mechanism to adaptively learn the weights. Taking drug i as an example, the impact of neighbor j on i can be calculated by Eq. 8.

$$\alpha_{ij}=softmax\left(e_{ij}\right)=\frac{\exp\left(e_{ij}\right)}{\Sigma_{k\in N_{i}}\;\exp\left(e_{ik}\right)}$$ (8)

N indicates the neighbors of node i . The attention coefficient $\alpha_{ij}$ is obtained from $e_{ij}$ normalized by the softmax function, and $e_{ij}$ can be calculated using Eq. 9.

$$e_{ij}=LeakyReLU\left(w^{T}\left[Wh_{i}∥Wh_{j}\right]\right)$$ (9)

where ∥ indicates the concatenation operation, T means transpose, and W denotes the parameter matrix. $h_{i}$ , and $h_{j}$ indicate drug i and j feature vectors, respectively. Similarly, we can obtain the attention score of other neighboring nodes on it. Finally, we weigh the average to update the node representation, which is formulated as Eq. 10.

$$h_{i}=\sigma \left(\underset{j\in N_{i}}{\sum }\alpha_{ij}Wh_{j}\right)$$ (10)

#### View-level attention mechanism

We use view-level attention to assign the appropriate weights based on their contribution to the prediction task, which can help to reduce noise and achieve more efficient integration.

We first calculate the attention coefficients of three graph embeddings. For simplicity, we denote them by $\varepsilon_{a}$ , $\varepsilon_{k}$ , and $\varepsilon_{d}$ , whose subscripts indicate the first letter of the related view, as shown in Eq. 11.

$$\left(\varepsilon_{a},\varepsilon_{d},\varepsilon_{k}\right)=att\left({\;Z}_{adj}\;,Z_{diff}\;,Z_{knn}\;\right)$$ (11)

Taking the calculation process of $\varepsilon_{a}$ as an example, drug i in adjacency graph embedding $Z_{adj}$ can be denoted as $Z_{adj}^{i}$ . We first apply a nonlinear transformation and multiply by the shared attention vector q to obtain its attention value $w_{adj}^{i}$ , as shown in Eq. 12.

$${w_{adj}^{i}=q}^{T}∙\tanh\left(w∙{\left(\;Z_{adj}^{i}\right)}^{T}+b\right)$$ (12)

where w is the trainable weight matrix and b is the bias vector. After the same operation, the attention values $w_{diff}^{i}$ and $w_{knn}^{i}$ of the drug i in embedding $Z_{diff}$ and $Z_{knn}$ can be obtained. Then, we use the softmax function to regularize the attention values to obtain its final weight coefficients, as shown in Eq. 13.

$$\varepsilon_{a}^{i}=softmax\left(w_{adj}^{i}\right)=\frac{\exp\left(w_{adj}^{i}\right)}{\exp\left(w_{adj}^{i}\right)+\exp\left(w_{diff}^{i}\right)+\exp\left(w_{knn}^{i}\right)}$$ (13)

Similarly, we can obtain the attention scores $\varepsilon_{d}^{i}$ , and $\varepsilon_{k}^{i}$ for the other two views. For all drugs, the attention scores can be collected together and expressed as $\varepsilon_{a}=\left[\varepsilon_{a}\right]$ , $\varepsilon_{d}=\left[\varepsilon_{d}\right]$ , and $\varepsilon_{k}=\left[\varepsilon_{k}\right]$ , which are formally denoted as $\varepsilon_{a}=diag\left(\varepsilon_{a}\right)$ , $\varepsilon_{d}=diag\left(\varepsilon_{d}\right)$ , and $\varepsilon_{k}=diag\left(\varepsilon_{k}\right)$ after diagonalization. Finally, the unified drug embedding representations Z  were obtained as shown in Eq. 14.

$$Z=\varepsilon_{a}∙Z_{adj}+\varepsilon_{d}∙Z_{diff}+\varepsilon_{k}∙Z_{knn}$$ (14)

### Loss optimization

The candidate drug pairs i , and j in the unified embedding Z are denoted by Ф(di) and Ф(dj) , respectively. We combine them in four different ways to represent the drug pair vector, as shown in Table 2. The symbol ⊙ indicates the element-wise product, while the symbol ⊕ indicates the concatenation operation.

**TABLE 2:** Four different combinations of drug pairs.

Combination method	Dimensionality	Description
Average	d	$Ф\left(di,dj\right)=\frac{1}{2}\left[Ф\left(di\right)+Ф\left(dj\right)\right]$
Hadamard	d	Ф(di,dj)=Ф(di)⊙Ф(dj)
L1 norm	d	Ф(di,dj)=|Ф(di)−Ф(dj) |
Concatenation	2d	Ф(di,dj)=Ф(di) ⊕ Ф(dj)

For the prediction task of drug reactions, this paper combines cross-entropy loss $l_{ce}$ with reconstruction loss $l_{re}\;$ to constrain the model. We decode from the learned embedding to obtain the reconstruction loss, which is formulated as Eq. 15.

$$l_{re}=\overset{N}{\underset{i=1}{\sum }}∥Xi-\hat{X}i∥$$ (15)

where N is the number of drugs, and Xi and $\hat{X}i$ denote the raw and reconstructed features of drug i , respectively. For the multiclass classification task, we use the cross-entropy loss function, which is defined as Eq. 16.

$$l_{ce}=\sum_{\;l\in L}\overset{c}{\underset{i=1}{\sum }}Y_{\;li}\ln{\hat{Y}}_{\;li}$$ (16)

where L denotes the drug pair training set and c is the set of drug reaction types. $\hat{Y}\in R^{L\times c}$ and $Y\in R^{L\times c}\;$ denote the predicted label set and the true label set, respectively.

## Experiments

### Experimental setup

#### Dataset

The experimental data in this paper come from DDIMDL (Deng et al., 2020), which contains 572 drugs with four different types of features: chemical substructures, targets, enzymes, and pathways. There are also 37,264 drug pairs with 65 types of reaction events. According to the DDIMDL experiment, we used the three most effective features, i.e., chemical substructure, target, and enzyme for experiments.

#### Baselines

The following state-of-the-art methods were used to evaluate the performance of MFDA.

- Classic classifier: These methods are traditional supervised learning methods. The unified feature vector X is directly treated as drug features and input into the logistic regression (LR) (DeStefano, 1990), and random forest (RF) (Breiman, 2001) for training.
- DNN: We reproduced Lee’s idea (Lee et al., 2019) with a deep neural network (DNN). The drug pair vectors of the three features are concatenated and fed into the DNN classifier.
- DeepDDI^1^: DeepDDI (Ryu et al., 2018) is the first model to apply deep learning to drug reaction events. It uses the chemical substructure similarity of the drugs as the input and predicts the interaction type through a deep learning model. In this work, we adjusted the output to 65 classes to accommodate multitype DDI prediction.
- DDIMDL^2^: DDIMDL (Deng et al., 2020) optimizes the DeepDDI (Ryu et al., 2018) model and inputs three drug-related features of chemical substructure, target, and enzyme into the three constructed sub-models for training.
- LINE: LINE (Tang et al., 2015) considers the first-order similarity and second-order similarity between nodes.
- HOPE: HOPE (Ou et al., 2016) is a factorization method that maintains high-order proximity by reconstructing the adjacency matrix.
- Node2Vec: Node2vec (Grover and Leskovec, 2016) employs a biased random walking strategy to obtain a sequence of neighbor nodes and then inputs them into the skip-gram model to get the embedding of nodes
- SDNE: SDNE (Wang et al., 2016) improves the LINE method. It utilizes deep AE models to optimize both first- and second-order similarities instead of optimizing them separately.
- GAE: GAE (Kipf and Welling, 2016) adopts an encoder-decoder structure to learn embedding representations and train the model by minimizing the reconstruction loss.
- SkipGNN^3^: SkipGNN (Huang et al., 2020) aggregates messages from two-hop neighbors and immediate neighbors in the interaction network.
- RANEDDI^4^: RANEDDI (Hui et al., 2021) considers multiple relationships between drugs. It uses the knowledge graph-based approach RotatE (Sun et al., 2019) to learn initial drug embeddings, which are fed into the relationship-aware network to predict multiple DDI reactions.
- DPDDI^5^: To be fair, this experiment incorporates attribute feature information based on the original DPDDI (Feng et al., 2020) and then uses graph convolutional networks (GCNs) to learn the drug representation for prediction.
- DANE^6^: DANE (Gao and Huang, 2018) not only captures potential high nonlinearity in topology and attributes but also maintains first-order and high-order the proximities in the original network.
- AM-GCN^7^: AM-GCN (Wang et al., 2020) constructs KNN graphs from the node feature space; and network topology graphs from the interaction relationship of nodes. Then, the attention mechanism is used to adaptively integrate deep correlation information between topological structures and node features.
- MGCCN: MGCCN (Wang et al., 2022) builds three graphs from drug relationships and uses multiple parallel convolutional layers for each graph to learn topological representation. Finally, the multigraph attention module is used to obtain a unified node representation.

Reimplement details: the above comparison methods can be classified into four groups, and the detailed settings refer to Table 3. To ensure fairness, the embedding results learned by the model are saved and uniformly classified by using a DNN classifier. Note that the embedding learning methods (LINE, HOPE, Node2vec, SDNE, GAE) can be re-implemented in the BioNEV framework (Yue et al., 2020).

**TABLE 3:** Baseline method settings.

Type	Method	X	$A_{adj}$	$A_{diff}$	$A_{knn}$
Feature-based	LR,RF,DNN,DeepDDI,DDIMDL	✓			
Network-based	LINE,HOPE,Node2vec,SDNE,GAE, SkipGNN, RANEDDI		✓		
Feature and Network based	DPDDI, DANE	✓	✓		
Multiview-based	AM-GCN		✓		✓
	MGCCN		✓	✓	✓
Proposed	MFDA	✓	✓	✓	✓

X : feature matrix $A_{adj}:$ adjacency graph $A_{diff}:$ diffusion graph $A_{knn}:$ knn graph.

#### Evaluation metrics

To comprehensively evaluate the performance of MFDA, we used six evaluation metrics, including the accuracy (ACC), area under the precision-recall curve (AUPR), area under the receiver operating characteristic (ROC) curve (AUC), F1, Precision (Pre), and Recall. We use micro metrics for AUPR and AUC and macro metrics for the other metrics (Pre_macro, F1_macro, Recall_macro).

#### Parameter and evaluation settings

The maximum iteration number is set to 1,000, and we selected the Adam optimizer with a learning rate of 0.003 to optimize MFDA.

Meanwhile, we applied fivefold cross-validation in our experiments and randomly divided all DDI pairs into five subsets. The final score was calculated by taking the average of the five rounds of output. To avoid overfitting, we used the early-stopping strategy, which automatically terminated training after 20 epochs if no improvement was observed. Regarding the settings of the comparison method parameters, they all follow the original paper. The experiment was conducted on Windows Server 2016 Datacenter, which was configured with Intel Xeon Processor (Skylake, IBRS), 2.3 GHz, 32 CPUs. It was running on python 3.8 and the total running time was about 1551.76 s.

## Results and analysis

The results of the comparison experiments in Table 4 show that RANEDDI has the highest score on ACC, which may be due to the KG-based approach used. The KG-based approach can capture the relationships between multiple entities and obtain rich structural and semantic information. And Node2Vec scored highest on AUC_micro, which was due to its biased sampling that integrated structural features obtained by depth and breadth search. However, MFDA performed best overall, outperforming RANEDDI by 12.68, 9.71, and 13.97% in F1_macro, Pre_macro, and Recall_macro metrics, respectively, and by 7.13, 5.09, and 8.23% over Node2vec.

**TABLE 4:** Performance of our model against competitive approaches.

Type	Method	ACC		AUC_PR_micro	AUC_ micro	F1_macro	Pre_macro	Recall_macro
Feature-based	LR		0.721	0.785	0.993	0.306	0.504	0.254
	RF		0.772	0.846	0.995	0.481	0.713	0.408
	DNN		0.880	0.913	0.996	0.722	0.805	0.703
	DeepDDI		0.837	0.890	0.996	0.685	0.728	0.661
	DDIMDL		0.885	0.921	0.998	0.759	0.847	0.718
Network-based	LINE		0.883	0.949	0.999	0.750	0.774	0.746
	HOPE		0.902	0.962	0.999	0.762	0.794	0.749
	Node2vec		0.907	**0.966**	0.999	0.780	0.806	0.770
	SDNE		0.777	0.848	0.995	0.466	0.579	0.441
	GAE		0.784	0.860	0.996	0.491	0.587	0.454
	SkipGNN (2020)		0.758	0.863	0.855	0.755	0.773	0.759
	RANEDDI (2021)		**0.967**	0.952	0.999	0.724	0.759	0.713
Feature and Network based	DPDDI (2020)		0.825	0.900	0.997	0.655	0.723	0.643
	DANE (2019)		0.871	0.937	0.998	0.705	0.759	0.679
Multiview-based	AM-GCN(2020)		0.877	0.945	0.998	0.745	0.783	0.726
	MGCCN (2022)		0.899	0.961	0.999	0.802	0.820	0.796
Proposed	MFDA (GAT)		0.901	0.961	0.999	0.807	0.826	0.797
	MFDA (GAT&KNN)		0.905	0.964	0.999	0.822	0.833	0.820
	MFDA (ours)		0.902	0.963	**0.999**	**0.851**	**0.857**	**0.853**

The best results are shown in bold.

**FIGURE 3:**
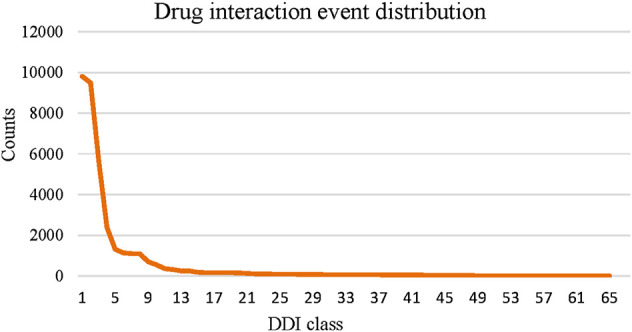
Statistics of DDI events.

Compared to the multiview-based methods, we can see that MFDA is better than the best MGCCN, the difference is that in each view, we use a cross-fusion strategy to deeply fuse drug features and topological information, rather than simply fusing with the GCN. Besides, the proposed MFDA has the best results compared to other view ablation variants, which demonstrated that integrating multiple views is beneficial to improve model performance. In addition, we observed that DDIMDL performed better in feature-based methods. A plausible explanation was that DDIMDL used three types of drug features and captured different heterogeneous information from them. However, the GAE, and SDNE performed poorly in the network-based methods. This can be attributed to the unbalanced distribution of multiple DDI events. For verification, we counted the DDI events involved in the experiment, as shown in Figure 3. The results showed that there was a sharp decline over 65 DDI class distributions, and the data were unevenly distributed. This could directly affect the overall performance of the model using GCN as the encoder.

### Ablation study

**FIGURE 4:**
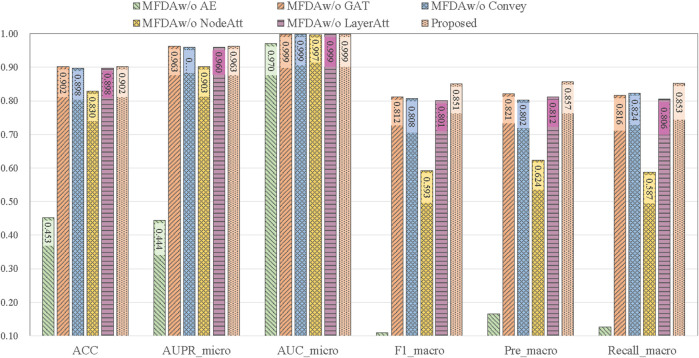
Results of ablation experiments.

To explore the influence of each module in the model, we set five ablation variants, as detailed in Table 5 and the results of the ablation experiments are shown in Figure 4.

- MFDAw/o AE: This variant has no AE component. The fusion coefficient alpha was set to 0, so the MFDA degenerated into a multilayer GAT model, capturing only the information of the network structure.
- MFDAw/o GAT: This variant has no GAT module. It inputs the embedding representation obtained by AE into the model for end-to-end training and uses only the feature information of the drug.
- MFDAw/o Convey: There is no convey operation to exchange information between layers. It combined the embeddings learned from two independent networks through an attention mechanism to obtain a unified embedding vector. This variant was designed to verify the importance of the convey operation.
- MFDAw/o NodeAtt: This variant does not use the node-level attention mechanism. It replaced GAT with GCN, which uniformly aggregated neighboring nodes. The rest of the settings are the same as MFDA.
- MFDAw/o LayerAtt: This variant does not use a view-level attention mechanism. When fusing three view embeddings, the final drug embedding is obtained by averaging method.

**TABLE 5:** Ablation variants settings.

	_AE	_GAT	_Convey	_ NodeAtt	_ LayerAtt
MFDA _w/o_ AE		✓			
MFDA_w/o_ GAT	✓				
MFDA _w/o_ Convey	✓	✓		✓	✓
MFDA _w/o_ NodeAtt	✓		✓		✓
MFDA_w/o_ LayerAtt	✓	✓	✓	✓	

The results in Figure 4 show that the ablation variants scored lower on all metrics compared to MFDA, indicating that each component of the model is functional. In addition, we note that the MFDA_w/o_ AE variant, which does not have the AE model to capture feature information, decreases significantly across all metrics, suggesting that feature information contributes the most to the prediction task, followed by the attention mechanism.

### Parameter sensitivity analysis

**FIGURE 5:**
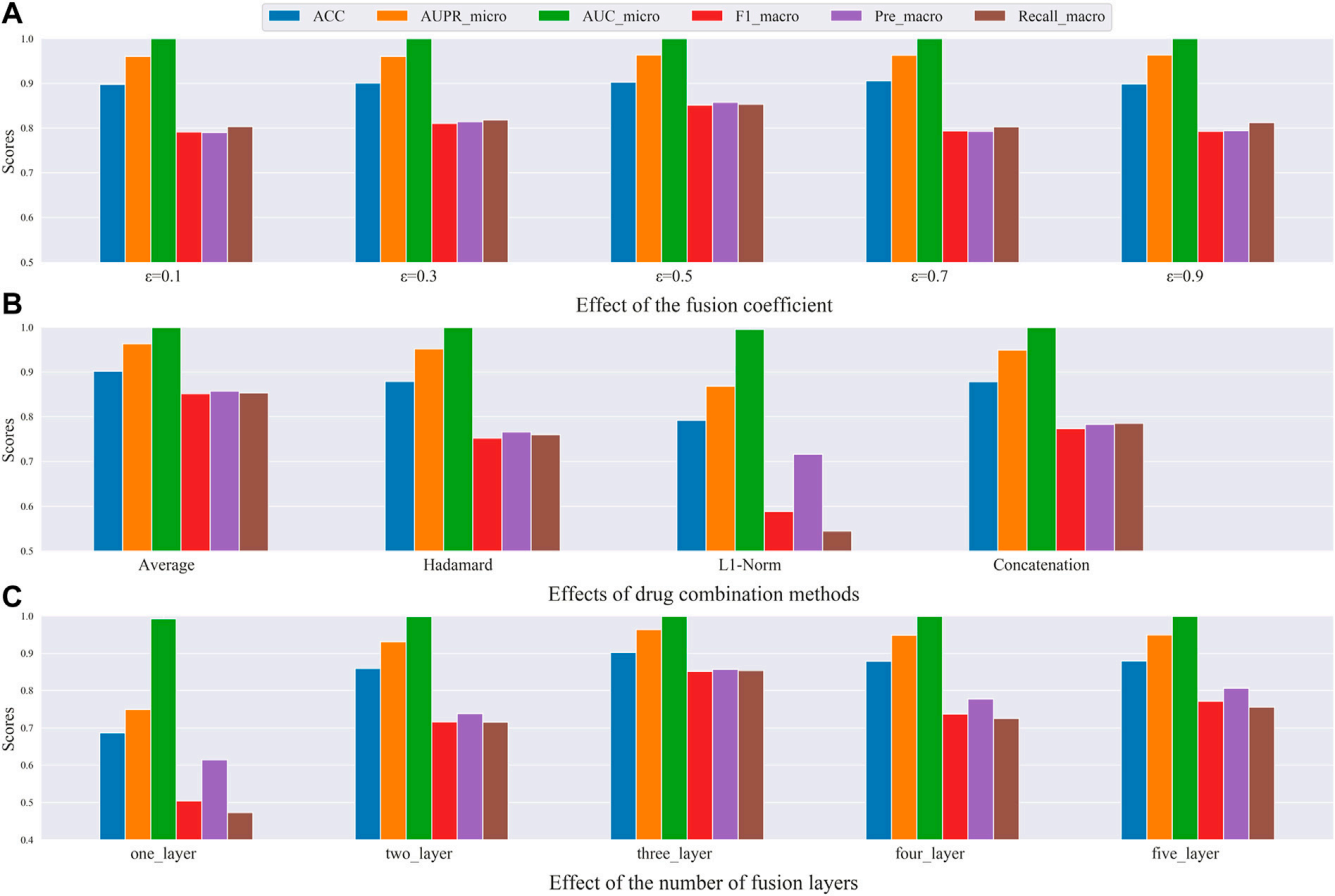
Results of ablation experiments. **(A)** Effect of fusion coefficient. **(B)** Effects of drug combination methods. **(C)** Effect of the number of fusion layers.

In this section, we investigate the effects of the fusion coefficient ε , the method of combining drug pairs, and the number of fusion layers on the experiment to find the best parameters. The results of the experiment are shown in Figure 5.

The fusion coefficient ε , which controls the weight of embeddings learned from the two types of networks. First, we fixed the other two parameters and observed the experimental results by varying ε in the range of {0.1, 0.3, 0.5, 0.7, 0.9}. The results from Figure 5A showed that when the fusion coefficient ε was set to 0.5, that is, when the GAT embedding was equally fused with the AE embedding, the best results were obtained.

Different methods of drug combination affect the meaning of the constructed drug-pair vectors. Here, we explore the effect of different combinations on the experimental results. The results in Figure 5B show that when the average method is applied, almost all metrics achieved the highest value.

To evaluate the fusion layer parameters, we varied layers one to five and the number of hidden layer nodes was empirically set to (2000, 512, 512, 256, 65). We fixed the number of neurons in the last hidden layer to 65, as there are 65 types of DDI events. Then, the number of layers was gradually increased to evaluate how the number of layers affects the experimental results. The results are shown in Figure 5C, where the model performance showed an increasing trend until it reached three levels, indicating that it incorporated more useful information. When the layer number is 3, all metrics reached the highest point. After that, all of them had a decreasing trend, indicating that too many layers may also incorporate noisy information, which instead reduced the model effect. Finally, we set the number of layers to 3 as a trade-off.

### Multitask analysis

**FIGURE 6:**
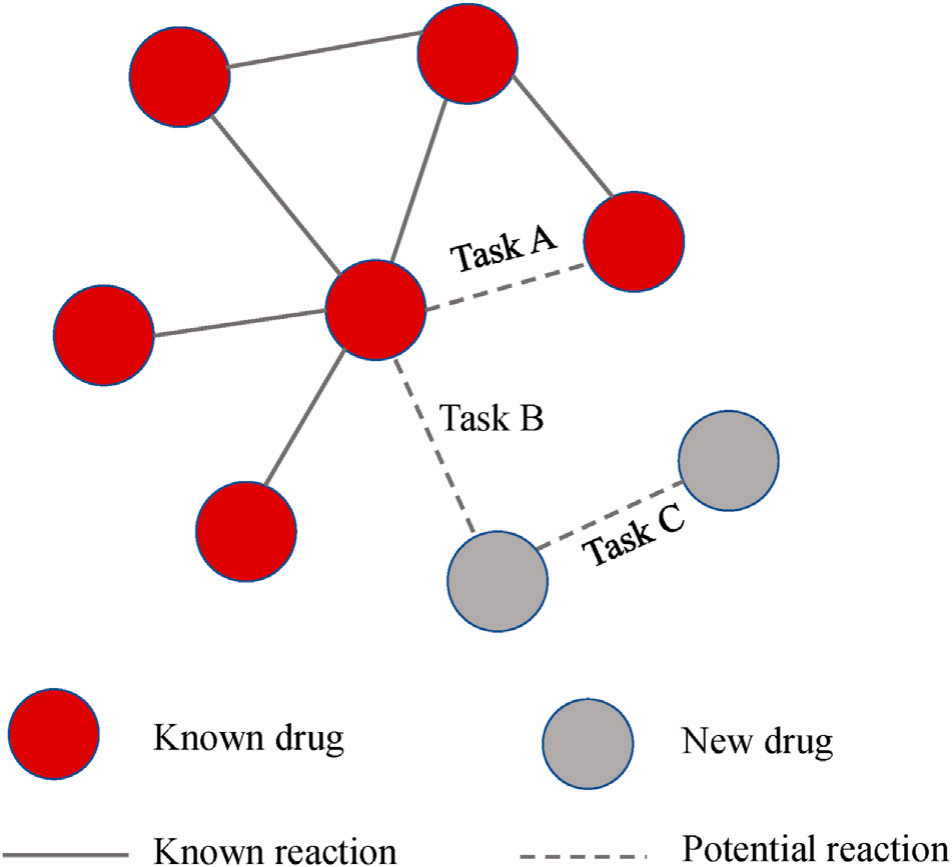
The relationship between the three tasks.

In general, we are more interested in the ability to predict reactions to new drugs. The previous experiment is equivalent to Task A in Figure 6, which evaluates the model’s ability to predict known drugs. While Task B and Task C were designed to assess the predictive ability of the new drug reactions. For clarity, the relationship between the three tasks is shown in Figure 6.

- Task A: We applied fivefold cross-validation and randomly split all drug pair vectors into five subsets. We trained models based on DDIs in the training set and then made predictions for DDIs in the test set. The evaluation score was the average of the output of the five rounds.
- Task B: Without using previous drug pairs, 572 drugs were randomly divided into five subsets. We used one of them as a test set to simulate drugs with no known interactions. The model was constructed on the training drugs and tested between the training drugs and testing drugs.
- Task C: This design is similar to Task B. The difference is that the model is tested between the testing drugs, which is equal to predicting two completely new drugs.

We selected multiple drug reaction methods (DeepDDI, DPDDI, DDIMDL) for comparison experiments on three tasks, and the experimental results are shown in Table 6.

**TABLE 6:** Results of the three types of tasks.

	Method	ACC	AUPR_micro	AUC_micro	F1_macro	Pre_macro	Recall_macro
Task A	DeepDDI	0.837	0.890	0.996	0.685	0.728	0.661
	DPDDI	0.784	0.860	0.996	0.491	0.587	0.454
	DDIMDL	0.885	0.921	0.998	0.759	0.847	0.718
	MFDA	**0.902**	**0.963**	**0.999**	**0.851**	**0.857**	**0.853**
Task B	DeepDDI	0.577	0.559	0.978	0.342	0.363	0.389
	DPDDI	0.520	0.518	0.965	0.214	0.259	0.227
	DDIMDL	0.642	0.656	0.971	0.446	0.561	0.432
	MFDA	**0.875**	**0.837**	**0.995**	**0.664**	**0.704**	**0.650**
Task C	DeepDDI	0.360	0.278	0.906	0.137	0.159	0.145
	DPDDI	0.311	0.259	0.925	0.045	0.051	0.048
	DDIMDL	0.408	0.364	0.951	0.159	0.248	0.145
	MFDA	**0.759**	**0.714**	**0.991**	**0.446**	**0.469**	**0.350**

The best results are shown in bold.

We observed a significant decrease in the scores of all models when predicted with new drugs. In particular, the AUPR_micro and F1_macro scores of DDIMDL in Task B dropped from 0.656 to 0.446, respectively, to 0.364, and 0.159 in Task C, respectively. However, the MFDA did not decrease drastically and always achieved optimal results. A possible explanation is that MFDA had a stable network structure, which constructed three views and was able to capture deep interaction information of drug features with the network topology under each view.

### Case study

In this section, we conduct case studies to validate the predictive ability of MFDA in practice. We performed experiments on 572 drugs that had 37,264 DDIs and 65 types of reaction events. The experimental goal is to predict the reaction events between the remaining drug-drug pairs. We pay attention to the top ten most common drug reaction events and recorded drug pair reactions with the highest scores in each reaction type. We used the interactions checker tool provided by DrugBank^8^ for verification. The experimental results are shown in Table 7. Seven DDI events can be identified out of the top ten events, which achieved good prediction accuracy. In addition, the predicted results were well-readable. For example, the predicted reaction of abemaciclib with astemizole was type “1″, which suggested that the metabolism of abemaciclib would be weaker when combined with astemizole. This specific type of predictive result could provide additional insight into the underlying mechanisms behind the drug reactions.

**TABLE 7:** Predicted results for the top 10 types of reaction events.

Drug1	Drug2	Predict label	Evidence	Description
Abemaciclib	Astemizole	1	Drugbank	The metabolism of abemaciclib can be decreased when combined with astemizole
Amiodarone	Morniflumate	2	Drugbank	The risk or severity of hyperkalemia can be increased when amiodarone is combined with morniflumate
Imatinib	Epinephrine	3	Drugbank	The serum concentration of epinephrine can be increased when it is combined with imatinib
Apalutamide	Azacitidine	4	Drugbank	Azacitidine may decrease the excretion rate of apalutamide which could result in a higher serum level
Conivaptan	Levosalbutamol	5	Drugbank	Conivaptan may increase the excretion rate of levosalbutamol which could result in a lower serum level and potentially a reduction in efficacy
Nabilone	Mepyramine	6	Drugbank	Nabilone may increase the central nervous system depressant (CNS depressant) activities of mepyramine
Atomoxetine	Methsuximide	7	N.A.	The risk or severity of QTc prolongation can be increased when atomoxetine is combined with methsuximide
Bosentan	Carisoprodol	8	N.A.	Bosentan may increase the hypotensive activities of carisoprodol
Aprepitant	Bivalirudin	9	N.A.	The metabolism of aprepitant can be increased when combined with bivalirudin
Diltiazem	Droxidopa	10	Drugbank	Droxidopa may decrease the antihypertensive activities of diltiazem

**FIGURE 7:**
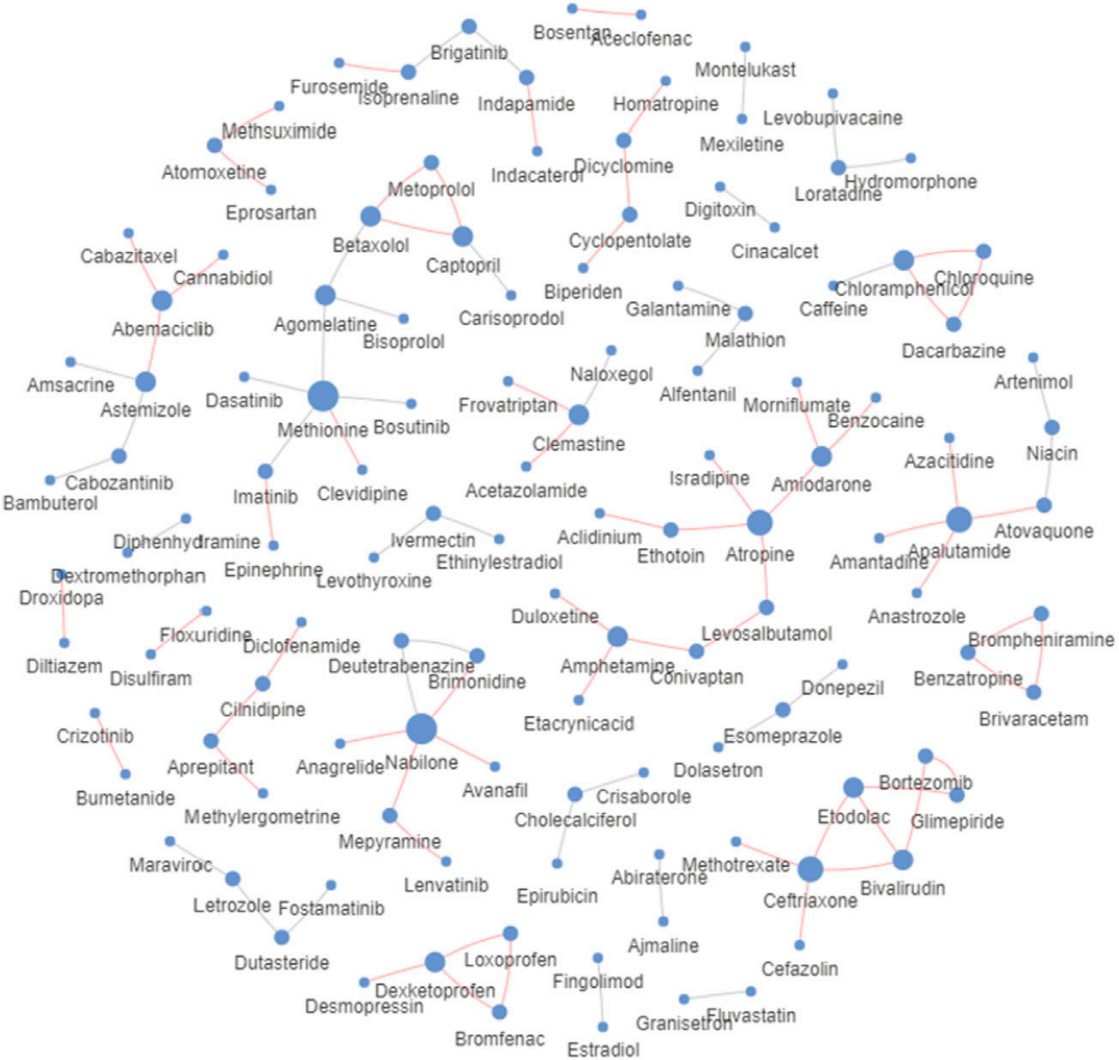
Validation of the predicted results for the top 100 drug pairs.

Furthermore, we selected the top 100 drug pairs with the highest scores and then checked up evidence in DrugBank. The results were organized into a relational graph (Figure 7) for presentation, where confirmed reactions were represented by red edges, otherwise by gray edges, and node size was related to the number of reactions involved. Statistically, 62% of the interactions have been confirmed. This case shows that MFDA is promising for predicting unknown reactions between drugs.

### Analysis of attention mechanism

**FIGURE 8:**
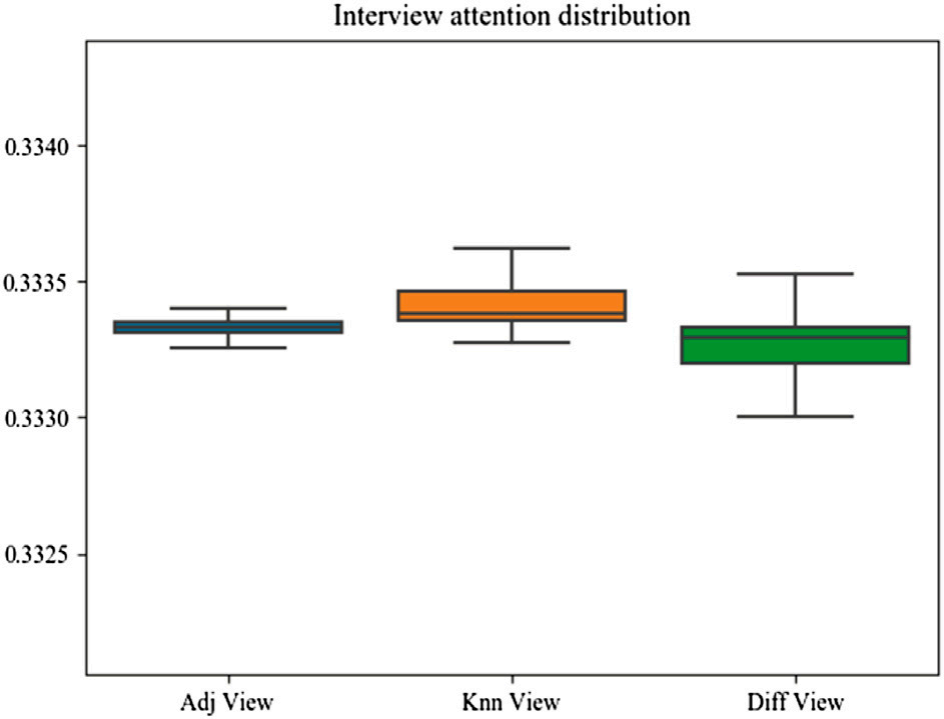
View-level attention distribution.

To overcome the lack of interpretability of graph neural networks, we predicted specific reaction events, which made the results more readable. Furthermore, we used a dual-level attention mechanism to provide interpretability for DDI. We first output three view-level attention scores as shown in Figure 8. From the attention distribution, we found that the KNN graph contributed more to drug prediction. The KNN graph revealed similarities in the drug feature space, which was in line with the conclusion drawn from the previous ablation experiments that drug feature information would be important.

**FIGURE 9:**
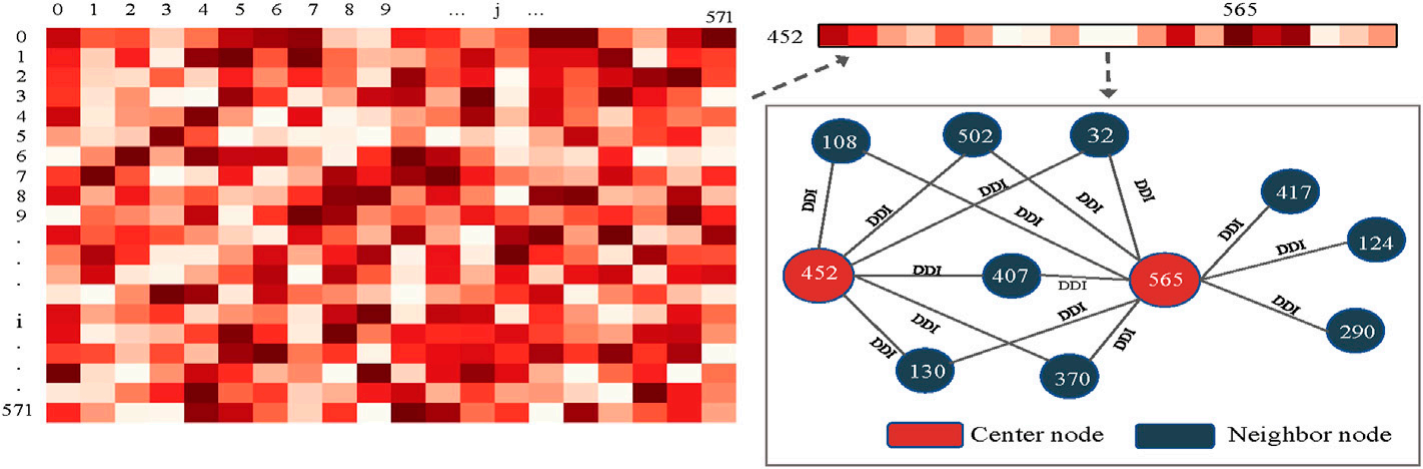
Node-level attention distribution and interpretation.

For further analysis, we chose to investigate node-level attention under the KNN view with a high importance score. Specifically, we saved the last layer of node-level attention scores under the KNN view and plotted it as a heatmap, as shown in Figure 9. We randomly selected a drug node 452 (Calcidiol) and found that it has a large correlation score with node 565 (Calcipotriol). They did not belong to the same type of drug, but we found six common neighbors between drug nodes 452 and 565 by analysing their relationships in known drug pair reactions. To make it clear, we used Echart^9^ for display. The two nodes shared many common neighbors, so there was a strong connection between the two nodes. To sum up, the dual-level attention mechanism allows us to know what view is the most important, and the relationship between neighbors. This can facilitate the understanding of drug reaction prediction.

## Conclusion

We proposed a novel method MFDA for the prediction of multiple drug reaction events. MFDA integrated information from multiple views, which reduced the dependence on a single view and greatly improved the stability of the model. In addition, under each view, convey operations were applied to flexibly exchange feature information with topology information, which helped to fully capture interaction information and therefore obtained high-quality drug representations. Through extensive comparative experiments as well as prediction experiments for new drugs, MFDA achieved satisfactory performance compared to optimal baselines. In addition, the dual-level attention mechanism could also provide an interpretable prediction for drug reactions. In the future, we may extend MFDA to heterogeneous graphs and explore capturing high-level embedding representations using knowledge graph-based approaches.

## Data Availability

Publicly available datasets were analyzed in this study. This data can be found here: https://github.com/kk12321/SKjjdk-MFDA.

## Footnotes

1
https://bitbucket.org/kaistsystemsbiology/deepddi

2
https://github.com/YifanDengWHU/DDIMDL

3
https://github.com/kexinhuang12345/SkipGNN

4
https://github.com/DongWenMin/RANEDDI

5
https://github.com/NWPU-903PR/DPDDI

6
https://github.com/gaoghc/DANE

7
https://github.com/zhumeiqiBUPT/AM-GCN

8 Drugbank: https://go.drugbank.com/

9
https://echarts.apache.org/zh/index.html

## Abbreviations

ACC, Accuracy; AE, Autoencoder; AUC, Area under the receiver operating characteristic curve; AUPR, Area under the precision-recall curve; DDI, Drug‒drug interaction; GAT, Graph attention network; KNN, K nearest neighbors; LR, logistic regression.

## References

[B1] Blum A. Mitchell T. (1998). “Combining labeled and unlabeled data with co-training,” in Proceedings of the eleventh annual conference on Computational learning theory, 92–100. 10.1145/279943.279962

[B2] Breiman L. J. M. L. (2001). Random forest. Mach. Learn. 45, 5–32. 10.1023/a:1010933404324

[B3] Cheng F. Zhao Z. (2014). Machine learning-based prediction of drug-drug interactions by integrating drug phenotypic, therapeutic, chemical, and genomic properties. J. Am. Med. Inf. Assoc. 21 (2), e278–e286. 10.1136/amiajnl-2013-002512 PMC4173180 24644270

[B4] Costanzo M. VanderSluis B. Koch E. N. Baryshnikova A. Pons C. Tan G. (2016). A global genetic interaction network maps a wiring diagram of cellular function. Science. 353 (6306), aaf1420. 10.1126/science.aaf1420 27708008 PMC5661885

[B5] Deng Y. Xu X. Qiu Y. Xia J. Zhang W. Liu S. J. B. (2020). A multimodal deep learning framework for predicting drug-drug interaction events. Bioinformatics 36 (15), 4316–4322. 10.1093/bioinformatics/btaa501 32407508

[B6] DeStefano J. J. (1990). “Logistic regression and the Boltzmann machine,” in International joint conference on neural networks, 199–204. 10.1109/ijcnn.1990.137845

[B7] Dong J. Zhao M. Liu Y. Su Y. Zeng X. (2022). Deep learning in retrosynthesis planning: Datasets, models and tools. Brief. Bioinform 23 (1), bbab391. 10.1093/bib/bbab391 34571535

[B8] Feng Y.-H. Zhang S.-W. Shi J.-Y. (2020). Dpddi: A deep predictor for drug-drug interactions. BMC Bioinforma. 21 (1), 419–515. 10.1186/s12859-020-03724-x PMC7513481 32972364

[B9] Gao H. Huang H. (2018). “Deep attributed network embedding,” in Twenty-seventh international joint conference on artificial intelligence (IJCAI). 10.24963/ijcai.2o18/46710.24963/ijcai.2018/467

[B10] Gottlieb A. Stein G. Y. Oron Y. Ruppin E. Sharan R. (2012). Indi: A computational framework for inferring drug interactions and their associated recommendations. Mol. Syst. Biol. 8, 592. 10.1038/msb.2012.26 22806140 PMC3421442

[B11] Grover A. Leskovec J. (2016). “node2vec,” in The 22nd ACM SIGKDD international conference. 10.1145/2939672.2939754

[B12] Huang H. Li J. Hu H. (2017). “Accelerated attributed network embedding,” in Proceedings of the 2017 SIAM international conference on data mining (SDM), 633–641. 10.1137/1.9781611974973.71

[B13] Huang K. Xiao C. Glass L. Zitnik M. Sun J. (2020). SkipGNN: Predicting molecular interactions with skip-graph networks. Sci. Rep. 10 (11), 21092–21116. 10.1038/s41598-020-77766-9 33273494 PMC7713130

[B14] Kang L.-P. Lin K.-B. Lu P. Yang F. Chen J.-P. (2022). Multitype drug interaction prediction based on the deep fusion of drug features and topological relationships. PloS one 17 (8), e0273764. 10.1371/journal.pone.0273764 36037188 PMC9423685

[B15] Kastrin A. Ferk P. Leskošek B. (2018). Predicting potential drug-drug interactions on topological and semantic similarity features using statistical learning. PloS one 13 (5), e0196865. 10.1371/journal.pone.0196865 29738537 PMC5940181

[B16] Kip F T. N. Welling M. (2017). “Semi-supervised classification with graph convolutional networks,” in International conference on learning representations. 10.48550/arXiv.1609.02907

[B17] Kipf T. N. Welling M. (2016). “Variational graph auto-encoders,” in Conference on neural information processing systems. 10.48550/arXiv.1611.07308

[B18] Kovács I. Luck K. Spirohn K. Wang W. Pollis D. K. Schlabach S. (2018). Network-based prediction of protein interactions. Cold Spring Harb. Lab. (1). 10.1101/275529 PMC6423278 30886144

[B19] Lee G. Park C. Ahn J. (2019). Novel deep learning model for more accurate prediction of drug-drug interaction effects. BMC Bioinforma. 20 (1), 415. 10.1186/s12859-019-3013-0 PMC6685287 31387547

[B20] Li L. Huang H. Fu Y. Wang J. Wu Z. Ru J. (2015). Large-scale exploration and analysis of drug combinations. Bioinformatics 31 (12), 2007–2016. 10.1093/bioinformatics/btv080 25667546

[B21] Lyu T. Gao J. Tian L. Li Z. Zhang P. Zhang J. (2021). “Mdnn: A multimodal deep neural network for predicting drug-drug interaction events,” in Proceedings of the thirtieth international joint conference on artificial intelligence, 3536–3542. 10.24963/ijcai.2021/487

[B22] Meng Y. Lu C. Jin M. Xu J. Zeng X. Yang J. (2022). A weighted bilinear neural collaborative filtering approach for drug repositioning. Brief. Bioinform 23 (2), bbab581. 10.1093/bib/bbab581 35039838

[B23] Ou M. Cui P. Pei J. Zhang Z. Zhu W. (2016). “Asymmetric transitivity preserving graph embedding,” in Proceedings of the 22nd ACM SIGKDD international conference on Knowledge discovery and data mining, 1105–1114. 10.1145/2939672.2939751

[B24] Pan X. Lin X. Cao D. Zeng X. Yu P. S. He L. (2022). “Deep learning for drug repurposing: Methods, databases, and applications,” in Wiley interdisciplinary reviews: Computational molecular science, 12, e1597. 10.1002/wcms.1597 WIREs Comput. Mol. Sci.

[B25] Rohani N. Eslahchi C. (2019). Drug-drug interaction predicting by neural network using integrated similarity. Sci. Rep. 9 (1), 13645–13711. 10.1038/s41598-019-50121-3 31541145 PMC6754439

[B26] Ryu J. Y. Kim H. U. Lee Y. L. (2018). Deep learning improves prediction of drug-drug and drug-food interactions. Proc. Natl. Acad. Sci. U. S. A. 115, E4304. 10.1073/pnas.1803294115 29666228 PMC5939113

[B27] Song B. Luo X. Luo X. Liu Y. Niu Z. Zeng X. (2022). Learning spatial structures of proteins improves protein-protein interaction prediction. Brief. Bioinform 23 (2), bbab558. 10.1093/bib/bbab558 35018418

[B28] Sun W. Sanderson P. E. Zheng W. (2016). Drug combination therapy increases successful drug repositioning. Drug Discov. Today 21 (7), 1189–1195. 10.1016/j.drudis.2016.05.015 27240777 PMC4907866

[B29] Sun Z. Deng Z. H. Nie J. Y. Tang J. (2019). “RotatE: Knowledge graph embedding by relational rotation in complex space,” in The international conference on learning representations. 10.48550/arXiv.1902.10197

[B30] Tang J. Qu M. Wang M. Zhang M. Yan J. Mei Q. (2015). “Line,” in Proceedings of the 24th international conference on world wide web, 1067–1077. 10.1145/2736277.2741093

[B31] Tatonetti N. P. Denny J. C. Murphy S. N. Fernald G. H. Krishnan G. Castro V. (2011). Detecting drug interactions from adverse-event reports: Interaction between paroxetine and pravastatin increases blood glucose levels. Clin. Pharmacol. Ther. 90, 133–142. 10.1038/clpt.2011.15910.1038/clpt.2011.83 21613990 PMC3216673

[B32] Vilar S. Harpaz R. Uriarte E. Santana L. Rabadan R. Friedman C. (2012). Drug-drug interaction through molecular structure similarity analysis. J. Am. Med. Inf. Assoc. 19 (6), 1066–1074. 10.1136/amiajnl-2012-000935 PMC3534468 22647690

[B33] Wang B. Wang Y. He X. Hu Y. Yin B. J. I. S. P. (2022). Multi‐graph convolutional clustering network. IET Signal Process. 16, 650–661. 10.1049/sil2.12116

[B34] Wang D. Cui P. Zhu W. (2016). “Structural deep network embedding,” in Proceedings of the 22nd ACM SIGKDD international conference on Knowledge discovery and data mining, 1225–1234. 10.1145/2939672.2939753

[B35] Wang X. Zhu M. Bo D. Cui P. Shi C. Pei J. (2020). “Am-gcn: Adaptive multi-channel graph convolutional networks,” in Proceedings of the 26th ACM SIGKDD International conference on knowledge discovery & data mining, 1243–1253. 10.1145/3394486.3403177

[B36] Wang Y. Min Y. Chen X. Wu J. (2021). “Multi-view graph contrastive representation learning for drug-drug interaction prediction,” in WWW '21: The web conference 2021. 10.1145/3442381.3449786

[B37] Yan C. Duan G. Zhang Y. Wu F.-X. Pan Y. Wang J. (2019). Idnddi: An integrated drug similarity network method for predicting drug-drug interactions, 89–99. 10.1007/978-3-030-20242-2_8

[B38] Yan C. Duan G. Zhang Y. Wu F. X. Pan J. Wang J. (2020). Predicting drug-drug interactions based on integrated similarity and semi-supervised learning. IEEE/ACM Trans. Comput. Biol. Bioinform 99, 1. 10.1109/TCBB.2020.2988018 32310779

[B39] Yan X. Yin P. Wu X. Han J. (2021). Prediction of the drug-drug interaction types with the unified embedding features from drug similarity networks. Front. Pharmacol. 12. 10.3389/fphar.2021.794205 PMC8721167 34987405

[B40] Yu H. Mao K. T. Shi J. Y. Huang H. Chen Z. Dong K. (2018). Predicting and understanding comprehensive drug-drug interactions via semi-nonnegative matrix factorization. BMC Syst. Biol. 12 (1), 14. 10.1186/s12918-018-0532-7 29671393 PMC5907306

[B41] Yu Y. A. Dong W. M. Shi B. (2022). Raneddi: Relation-aware network embedding for drug-drug interaction prediction. Inf. Sci. 582, 167–180. 10.1016/j.ins.2021.09.008

[B42] Yu Y. Huang K. Zhang C. Glass L. M. Sun J. Xiao C. J. B. (2021). SumGNN: Multi-typed drug interaction prediction via efficient knowledge graph summarization. Bioinformatics 37 (18), 2988–2995. 10.1093/bioinformatics/btab207 PMC10060701 33769494

[B43] Yuan J. Yu H. Cao M. Xu M. Xie J. Wang C. (2021). “Semi-supervised and self-supervised classification with multi-view graph neural networks,” in Proceedings of the 30th ACM international conference on information & knowledge management, 2466–2476. 10.1145/3459637.3482477

[B44] Yue X. Wang Z. Huang J. Parthasarathy S. Moosavinasab S. Huang Y. (2020). Graph embedding on biomedical networks: Methods, applications and evaluations. Bioinformatics 36 (4), 1241–1251. 10.1093/bioinformatics/btz718 31584634 PMC7703771

[B45] Zeng X. Tu X. Liu Y. Fu X. Su Y. (2022). Toward better drug discovery with knowledge graph. Curr. Opin. Struct. Biol. 72, 114–126. 10.1016/j.sbi.2021.09.003 34649044

[B46] Zhu J. Liu Y. Zhang Y. Li D. (2021). Attribute supervised probabilistic dependent matrix tri-factorization model for the prediction of adverse drug-drug interaction. IEEE J. Biomed. Health Inf. 25 (99), 2820–2832. 10.1109/jbhi.2020.3048059 33373310

[B47] Zitnik M. Agrawal M. Leskovec J. J. B. (2018). Modeling polypharmacy side effects with graph convolutional networks. Bioinformatics 34 (13), i457–i466. 10.1093/bioinformatics/bty294 29949996 PMC6022705

